# Balancing adaptive delivery and implementation fidelity over time: a longitudinal mixed-methods study of a community health worker–led adolescent nutrition intervention in rural Uganda

**DOI:** 10.1186/s43058-026-01072-0

**Published:** 2026-08-01

**Authors:** Thomas Buyinza, Edward Buzigi, Shuyan Liu, Justine Bukenya, Mary Mwanyika Sando, Till Bärnighausen, David Guwatudde, Rawlance Ndejjo

**Affiliations:** 1 Department of Epidemiology and Biostatistics, School of Public Health, Makerere University, Kampala, Uganda; 2 Iowa State University-Uganda Program, Center for Sustainable Rural Livelihoods, Kamuli, Uganda; 3 Department of Community Health and Behavioural Sciences, School of Public Health, Makerere University, Kampala, Uganda; 4 Department of Public Health and Nutrition, Faculty of Health Sciences, Victoria University, Kampala, Uganda; 5 Department of Psychiatry and Psychotherapy (Campus Charité Mitte), Charité – Universitätsmedizin, Berlin, Germany; 6 German Center for Mental Health (DZPG), Berlin, Germany; 7 Africa Academy for Public Health, Dares Salaam, Tanzania; 8 Heidelberg Institute of Global Health (HIGH), Medical Faculty and University Hospital, Heidelberg University, Heidelberg, Germany; 9 Department of Global Health and Population, Harvard T.H. Chan School of Public Health, Harvard University, Boston, USA; 10 Africa Health Research Institute (AHRI), Somkhele, Durban, South Africa; 11 Department of Disease Control and Environmental Health, School of Public Health, Makerere University, Kampala, Uganda; 12 Department of Preventive Medicine, College of Medicine, Korea University, Seoul, South Korea

**Keywords:** Adaptive delivery, Implementation fidelity, Community health workers, Adolescent nutrition, Consolidated framework for implementation research, Implementation research, Rural Uganda

## Abstract

**Background:**

Adolescents and young adults (AYAs) aged 10–24 years in sub-Saharan Africa experience low nutrition literacy and poor diet quality. Community health worker (CHW)-led interventions offer a delivery platform, yet little is known about how implementation proceeds under routine conditions or how fidelity is maintained alongside adaptive delivery adjustments. We assessed implementation fidelity over time and examined how contextual influences and supervision-guided adaptive delivery adjustments shaped delivery processes and supported preservation of core intervention functions during the nine-month implementation of a CHW-led intervention in Uganda.

**Methods:**

This longitudinal mixed-methods implementation study was nested within a nine-month randomized controlled trial in rural Eastern Uganda. Fidelity was assessed across early, mid, and late implementation using Carroll’s framework. Qualitative data were collected through repeated focus group discussions with CHWs, AYAs, and parents. Data were analysed using an inductive–deductive approach: adaptive delivery processes were identified inductively and organized and interpreted using the Consolidated Framework for Implementation Research. Quantitative and qualitative findings were integrated using joint displays to compare fidelity findings with qualitative contextual influences, supervision, and adaptive delivery adjustments.

**Results:**

Fidelity scores were high across implementation periods, with modest variation across domains. On a 1–5 scale, overall comparable fidelity scores were 4.18 (SD 0.12), 4.21 (SD 0.07), and 4.16 (SD 0.08) during early, mid, and late implementation, respectively. Adherence remained stable while quality of delivery was slightly higher during mid-implementation. Five adaptive delivery processes were identified: flexible scheduling and revisits; contextualized reinforcement of complex content; resource-sensitive counselling adjustment; workload-responsive micro-planning; and network-supported participant follow-up. Qualitative findings indicated that participant availability, comprehension challenges, household resource constraints, CHW workload, and participant mobility shaped delivery. Supervisory reflection and debrief provided a forum for reviewing challenges, sharing practical solutions, harmonizing delivery practices, and agreeing on context-responsive adjustments. These adjustments modified delivery forms, including visit timing, communication approaches, locally feasible examples, visit sequencing, and follow-up arrangements, while preserving core intervention functions.

**Conclusion:**

Adaptive delivery and implementation fidelity can be managed as interdependent implementation processes rather than competing priorities. Fidelity monitoring, supportive supervision, and guided problem-solving supported context-responsive delivery adjustments while supporting maintenance of core intervention functions.

**Trial registrations:**

The parent randomized controlled trial was prospectively registered with the Pan African Clinical Trials Registry (PACTR202406699274475) on 7 June 2024. https://pactr.samrc.ac.za/TrialDisplay.aspx?TrialID=30558.

**Supplementary Information:**

The online version contains supplementary material available at https://doi.org/10.1186/s43058-026-01072-0.

Contributions to the literature

- Provides longitudinal empirical evidence that adaptive delivery and implementation fidelity can be managed as interdependent implementation processes rather than competing priorities during routine delivery of a community health worker–led intervention.
- Identifies supportive supervision-mediated feedback as a process linking fidelity monitoring, reflective problem-solving, and adaptive delivery adjustments.
- Shows how prospective fidelity assessment and repeated qualitative data can be integrated to examine adaptive delivery processes in low-resource community health systems.
- Clarifies how core intervention functions can be preserved while delivery forms are adjusted to fit changing implementation conditions.

## Background

Adolescents and young adults (AYAs) have increased nutritional requirements during a critical period of physical, cognitive, and psychosocial development [1]. However, unhealthy and inadequate dietary patterns remain widespread globally, particularly in Sub-Saharan Africa (SSA), where structural constraints limit access to healthy foods [2–6]. In Uganda, low dietary diversity and inadequate fruit and vegetable intake are common among AYAs [7, 8]. These patterns are shaped by low nutrition literacy and broader socio-ecological influences, underscoring the need for contextually appropriate, household-level interventions that engage adolescents alongside their caregivers [9–12].

Community health workers (CHWs) play a central role in preventive health delivery in low- and middle-income countries, including Uganda, where they provide health education, counselling, and linkage to services at the household level [13–16]. In Uganda, CHWs ‒ commonly referred to as village health teams ‒ are lay community-based volunteers within the national health system [17], and are well positioned to reach adolescents, including those out of school. To address challenges in delivering adolescent-focused interventions through routine systems, a community-based adolescent nutrition education intervention was implemented through CHW platforms in Uganda as part of the ARISE-NUTRINT study [18, 19]. While embedding interventions within CHW systems enhances feasibility and sustainability [17, 20], it also exposes delivery to contextual constraints and changing community conditions that shape how interventions are implemented in practice [13, 21].

Effective implementation depends not only on intervention design but also on how interventions are delivered within complex, resource-constrained health systems [20, 22, 23]. CHW-led programs often face contextual challenges, including competing livelihood demands, variability in implementer capacity and motivation, sociocultural norms, seasonal disruptions, and limited supervision [22, 24, 25]. These conditions may require implementers to adjust delivery processes to sustain implementation. However, such adjustments need to be understood in relation to implementation fidelity, defined as the extent to which an intervention is delivered as intended [26].

Implementation fidelity may vary as implementation conditions change and implementers respond to emerging challenges, creating tension between maintaining adherence to core functions and allowing flexibility in delivery [26]. Unguided changes, particularly those affecting core intervention functions, may compromise fidelity, whereas adjustments to delivery forms may support contextual fit when core functions are preserved [27–29]. This distinction is important because fidelity and adaptive delivery adjustments are often treated as competing priorities, although they may be managed together when core functions are clearly specified.

Despite increasing recognition of the need to balance fidelity and flexibility, much of the adolescent nutrition literature in SSA has focused on intervention effectiveness, with limited attention to how delivery is adjusted in practice, how such adjustments are guided, and how core functions are maintained during routine implementation [30–35]. Implementation research has also often assessed determinants or fidelity at single time points, providing limited insight into how these processes evolve over time [36, 37]. This study assessed implementation fidelity over time and examined how contextual influences and supervision-guided adaptive delivery adjustments shaped delivery processes and supported preservation of core intervention functions during the nine-month implementation of a CHW-led intervention in rural Uganda.

## Methodology

### Study setting

The study was conducted in rural Mayuge District within the Iganga–Mayuge Health and Demographic Surveillance Site (HDSS) in Eastern Uganda [38]. This setting is predominantly rural and characterized by subsistence farming, seasonal livelihood activities, and limited dietary diversity among adolescents [8]. Households in this context often experience fluctuations in food availability and access due to agricultural cycles and economic constraints, which shape dietary behaviours [11].

### Study design

This was a longitudinal mixed-methods implementation study nested within a nine-month randomized controlled trial of a CHW–led adolescent nutrition education intervention, delivered from 15 May 2025 to 16 January 2026. The study combined prospective quantitative fidelity observations with repeated qualitative data collection to examine adaptive delivery processes and contextual influences across early (months 1–3), mid (months 4–6), and late (months 7–9) implementation. This design enabled examination of how adaptive delivery processes and implementation fidelity evolved over time under routine conditions.

### Overview of the intervention and implementation strategies

Details of the intervention package and delivery strategies have been published elsewhere [19]. The present paper summarizes the elements necessary for interpreting implementation fidelity and adaptive delivery adjustments. Briefly, the intervention was a community-based nutrition education package delivered by CHWs to adolescents and their caregivers at the household level. The intervention comprised nine sequential monthly modules covering balanced diet, sources and functions of essential vitamins and minerals, reduction of sugar-sweetened beverages, correction of food-related myths, and prevention of anaemia. Each module was delivered during a structured household session using a visual-aided flyer. Sessions followed a standardized flow guided by standard operating procedures (SOPs), incorporating explanation of key messages, participatory discussion, and caregiver engagement to support household-level application. Sessions lasted approximately 40–50 min, and flyers were left with households for ongoing reference.

For this study, core intervention functions were operationalized as delivery of the nine monthly modules, engagement of the target adolescent along the caretakers, use of visual-aided counselling materials, and reinforcement of key nutrition messages. Delivery forms referred to modifiable aspects of implementation, including visit timing, revisits after missed contacts, sequencing of household visits, locally relevant examples, communication approaches, and follow-up arrangements. Adaptive delivery adjustments were defined as modifications to these delivery forms made in response to contextual influences while seeking to preserve core intervention functions. Some flexibility, particularly in visit timing and revisits, was anticipated in the SOPs; other adjustments were identified during implementation through fidelity observations, and supportive supervision to sustain implementation amidst contextual constraints.

The main implementation strategies examined were SOP-guided delivery, supportive supervision, fidelity monitoring, feedback or debrief meetings, and guided problem-solving, described in alignment with recommendations for specifying implementation strategies [39]. Supportive supervision included observation of selected sessions using fidelity checklists and collective monthly debrief meetings with all ten CHWs. During debrief meetings, supervisors reviewed delivery progress, discussed observed challenges, and agreed on context-appropriate delivery adjustments to be used by CHWs where applicable, depending on the contextual influences encountered during household delivery.

### Study population and sampling

The qualitative component included CHWs who implemented the intervention, AYAs who received it, and parents or guardians who supported household-level application of intervention messages. Purposive sampling was guided by participants’ implementation roles and, for AYAs and parents or guardians, exposure to the intervention. All ten implementing CHWs were included in both the qualitative and quantitative fidelity components. All purposively selected participants consented to participate; none declined or dropped out.

AYA and parent or guardian participants were recruited at selected time points across early, mid, and late implementation to capture variation in age, sex, schooling status, and caregiving context. AYAs were grouped into three age categories: 10–14, 15–19, and 20–24 years. AYA and parent or guardian participants were approached at their households, with local council one leaders facilitating mobilization but not influencing participation. CHWs were approached directly as intervention implementers.

Across the study, 14 focus group discussions (FGDs) were conducted: six with CHWs, six with AYAs, and two with parents or guardians. CHW FGDs followed a panel design involving the same ten participants across repeated rounds to assess implementation learning over time. AYA and parent or guardian FGDs involved different participants at selected time points to capture diverse recipient experiences while reducing repeated-participation burden. Sampling and data collection proceeded iteratively until sufficient depth and variation in perspectives were achieved across participant groups. Overall, 60 unique participants contributed to the qualitative component.

### Conceptual framework

**Fig. 1:**
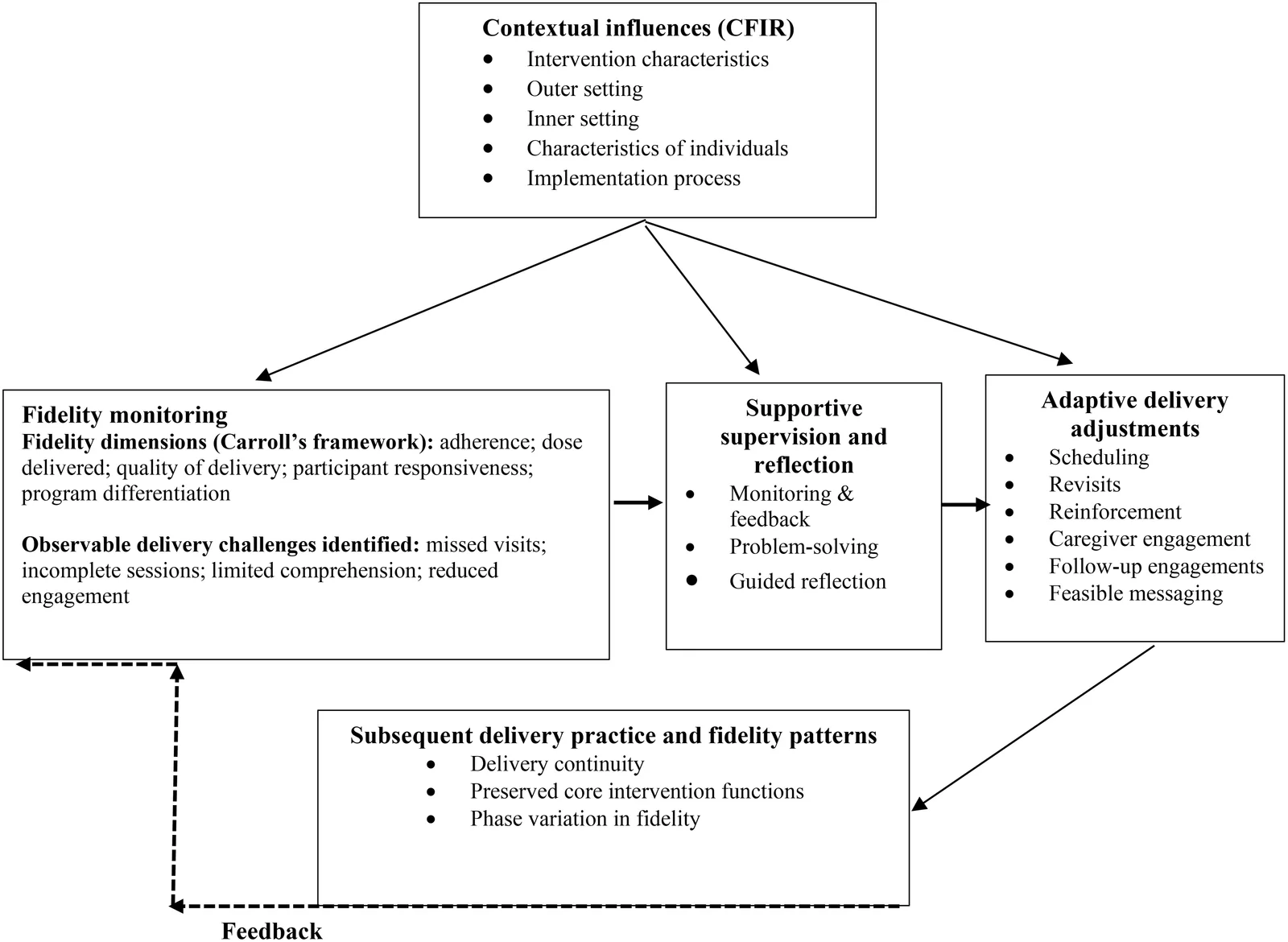
Conceptual framework of the supervision-mediated relationship between contextual influences, fidelity monitoring, supportive supervision, and adaptive delivery adjustments in a CHW-led adolescent nutrition intervention. CFIR, Consolidated Framework for Implementation Research

We synthesized an adaptation–fidelity feedback framework, drawing on the Consolidated Framework for Implementation Research (CFIR) [40], and Carroll’s fidelity framework [26], to guide the study (Fig. 1). The framework conceptualizes implementation as a dynamic process in which contextual influences shape delivery challenges, fidelity monitoring makes these challenges visible, and supportive supervision supports reflection, problem-solving, and adaptive delivery adjustments.

Contextual influences refer to broader conditions shaping implementation, such as household availability, school attendance, livelihood activities, household food resources, and competing CHW responsibilities. Implementation fidelity challenges refer to observable delivery issues, such as missed visits, incomplete sessions, limited comprehension, or reduced participant engagement. Thus, contextual influences represent implementation conditions, whereas fidelity challenges represent delivery-level manifestations of these conditions.

CFIR was used to organize and interpret contextual influences across intervention characteristics, outer setting, inner setting, characteristics of individuals, and implementation process domains, while Carroll’s framework guided fidelity assessment across adherence, dose delivered, quality of delivery, participant responsiveness, and program differentiation. Together, these frameworks guided analysis of implementation fidelity over time and interpretation of how contextual influences, supportive supervision, and adaptive delivery adjustments shaped delivery processes under routine conditions.

### Data collection procedures

#### Implementation fidelity

Implementation fidelity data were collected prospectively during routine intervention delivery from June 2025 to January 2026, covering the full nine-month implementation period. Fidelity assessments were conducted by a trained field implementation supervisor during routine, unannounced visits and were recorded contemporaneously. Each of the ten CHWs was directly observed once in each of the three implementation periods (months 1–3, 4–6, and 7–9) during real-time intervention delivery sessions, resulting in three observations per CHW. This enabled assessment of variation in fidelity scores across implementation periods under routine delivery conditions.

Fidelity was assessed using a structured observation checklist (Supplementary Material 1) aligned with the six components of Carroll et al.’s framework: adherence, dose delivered, does received, quality of delivery, participant responsiveness, and program differentiation [26]. Program differentiation was assessed from mid-implementation onward, after CHWs had delivered multiple modules and supervisors could more meaningfully assess whether intervention delivery remained distinct from other routine CHW activities. Each fidelity component was measured using multiple items rated on a 5-point Likert scale (1 = strongly disagree, 2 = disagree, 3 = neutral, 4 = agree, and 5 = strongly agree), based on predefined scoring criteria.

### Adaptive delivery processes

Qualitative data were collected through FGDs to explore adaptive delivery processes, contextual delivery challenges, supervision, and how delivery adjustments related to core intervention functions during implementation. All FGDs were guided by semi-structured discussion guides informed by CFIR (Supplementary Material 2). The guides were pretested among non-participants in Iganga district, a setting with socio-demographic characteristics similar to Mayuge [8, 16, 38]. The pretest assessed clarity, cultural appropriateness, and question flow, and feedback informed minor revisions to wording and sequencing. The finalized guides were translated into Lusoga, the primary local language, and contained open-ended questions tailored to each participant group.

FGDs were conducted in Lusoga by three research team members, two female and one male. All three held Master of Public Health degrees, were fluent in English and Lusoga, had prior experience in implementation research and qualitative data collection, and were serving as Research Associates at Makerere University School of Public Health (MakSPH) during the study. One researcher, TB, was also the field implementation supervisor. The researchers were not members of the study communities but were familiar with rural Eastern Ugandan contexts and adolescent nutrition issues. No personal relationships existed between researchers and participants before the study; however, TB interacted with CHWs during implementation supervision.

Each FGD involved approximately eight participants and lasted 60–90 min. Discussions were held in private, neutral venues such as schools or health centres, identified with support from local council one chairpersons. One researcher moderated, another managed audio recording and field notes, and the third (TB) supported probing on contextual influences and fidelity challenges, and guided adaptive delivery adjustment. FGDs were audio-recorded using digital voice recorders with informed consent. Field notes were taken during or immediately after discussions to capture non-verbal cues, contextual observations, and emerging issues. Only FGD participants and research team members were present.

At the start of each FGD, researchers introduced themselves as public health professionals conducting a study on intervention delivery and adaptive implementation. Participants were informed that participation was voluntary, responses would be confidential, and participation or responses would not affect their access to services, participation, or role in the intervention. Regular debriefing meetings were held between data collectors and two senior research team members, DG and JB, to review field experiences, provide feedback and further support address of emerging implementation and data collection challenges.

### Data management and analysis

#### Implementation fidelity

Quantitative fidelity data were entered, cleaned, and analysed using Stata version 17.0. Fidelity item scores were averaged within operational fidelity domains: adherence, dose delivered, dose received, quality of delivery, participant responsiveness, and program differentiation. Domain scores ranged from 1 to 5, with higher scores indicating stronger fidelity. Descriptive statistics, including means and standard deviations, were used to summarize fidelity domains across early, mid, and late implementation. Because program differentiation was not scored during early implementation, overall comparable fidelity scores across all three periods were calculated using domains available across all periods.

### Adaptive delivery processes

All audio-recorded discussions were transcribed verbatim in Lusoga and translated into English by two trained members of the research team. Transcripts were cross-checked by another team member, and an additional researcher independently reviewed selected transcripts against the original audio recordings to support translation accuracy. Transcripts were anonymized through removal of personal identifiers and assignment of pseudonyms. The transcripts were not returned to participants for comment, and participant checking of final themes was not conducted. Credibility was strengthened through field notes, triangulation across participant groups, and use of illustrative quotations.

The transcripts were analysed using NVivo version 12, following a hybrid inductive–deductive thematic approach, informed by Braun and Clarke’s phases of thematic analysis [41]. Three researchers independently read the transcripts to achieve data immersion and developed initial analytic memos. Open coding was first used to identify adaptive delivery processes, delivery forms modified, and whether core intervention functions were maintained. An initial codebook was developed and iteratively refined through team discussions. CFIR domains and selected constructs were then used to organize and interpret these processes in relation to contextual influences on implementation.

Each transcript was coded independently by at least two research team members. Coding discrepancies were resolved through consensus discussions involving all three analysts, with reference to analytic memos and original transcripts where necessary. Codes were grouped into subthemes and examined across implementation periods to describe variation over time. A summary coding tree is provided as Supplementary Material 3. Reporting of the qualitative component was guided by the Consolidated Criteria for Reporting Qualitative Research (COREQ) [42], and a completed checklist is provided as Supplementary Material 4.

To integrate quantitative and qualitative findings, domain-level fidelity scores from early, mid, and late implementation were interpreted alongside qualitative accounts collected during corresponding implementation periods. Integration focused on how delivery challenges, supervisory reflection, and adaptive delivery adjustments related to fidelity findings over time.

## Results

### Socio-demographic characteristics of participants

Results are presented in four parts: participant characteristics, implementation fidelity findings, adaptive delivery processes, and integrated evidence on how supervision-guided adaptive delivery adjustments related to fidelity findings across implementation periods.

Study participants were 60 in total, including 30 AYAs, 20 parents or guardians, and 10 CHWs (Table 1). Most AYAs (28/30) had attained primary or secondary education. All CHWs were aged 25 years and above and had at least primary education. Parents were predominantly married or cohabiting. Across participant groups, most individuals resided in households of 5–10 members (*n* = 38).

**Table 1:** Socio-demographic characteristics of participants (*n* = 60)

Characteristic	AYAs *n* = 30	Parents/Guardians *n* = 20	CHWs *n* = 10	Overall *n* = 60
**Age Group**				
10–14 years	10	0	0	10
15–19 years	12	0	0	12
20–24 years	8	0	0	8
25 years and above	0	20	10	30
**Schooling status (AYAs only)**				
In-school	15			15
Out of school	15			15
**Highest education attained**				
Primary and below	16	12	1	29
Secondary	12	6	8	26
Tertiary	2	2	1	5
**Household size**				
< 5 people	7	6	2	15
5–10 people	21	10	7	38
> 10 people	2	4	1	7
**Marital status**				
Single/never married	28	0	0	28
Married or cohabiting	2	16	10	28
Separated/Divorced	0	4	0	4
**Disability**				
Yes	2	0	0	2
No	28	20	10	58
**Employment**				
Formal	0	2	0	2
Informal	5	18	10	33
Not working	25	0	0	25

### Implementation fidelity scores across early, mid, and late implementation

Fidelity scores were generally high across implementation periods, with modest variation across domains (Table 2). On a 1–5 scale, the overall comparable fidelity score was 4.18 (SD 0.12) during early implementation, 4.21 (SD 0.07) during mid-implementation, and 4.16 (SD 0.08) during late implementation. Adherence remained stable across phases, while dose delivered and quality of delivery were slightly higher during mid-implementation. Program differentiation was high during mid and late implementation but was not assessed during early implementation.

**Table 2:** Fidelity domain scores across early, mid, and late implementation

Fidelity domain	No. of items	Early (months 1–3), Mean out of 5 (SD)	Mid (months 4–6),Mean out of 5 (SD)	Late (months 7–9), Mean out of 5 (SD)
Adherence	4	4.28 (0.32)	4.28 (0.28)	4.25 (0.17)
Dose delivered	4	4.08 (0.17)	4.23 (0.22)	4.15 (0.17)
Dose received	4	4.13 (0.32)	4.08 (0.33)	4.05 (0.20)
Quality of delivery	5	4.34 (0.16)	4.44 (0.13)	4.34 (0.23)
Participant responsiveness	4	4.10 (0.21)	4.05 (0.28)	4.03 (0.14)
Program differentiation	4	Not assessed	4.68 (0.21)	4.65 (0.27)
Overall comparable fidelity score*	**5 domains**	**4.18 (0.12)**	**4.21 (0.07)**	**4.16 (0.08)**

Note. Values are means and standard deviations (SD) across 10 CHW observations per implementation period. The overall comparable fidelity score was calculated as the mean of the five domains assessed across all three implementation periods, excluding program differentiation

### Adaptive delivery processes and preservation of core intervention functions

Five themes were identified, reflecting adaptive delivery processes across implementation and mapped to primary CFIR domains and constructs: flexible scheduling and revisits; contextualized reinforcement of complex content; resource-sensitive counselling adjustment; workload-responsive micro-planning; and network-supported participant follow-up (Table 3). For each theme, we present the original delivery expectation, delivery form modified, core intervention function intended to be preserved, and primary CFIR mapping.

Across the themes, adaptive delivery adjustments mainly involved changes to delivery forms, including visit timing, revisits, communication approaches, locally feasible examples, visit sequencing, and follow-up arrangements. These adjustments were described in relation to contextual influences encountered during household delivery and were discussed during supervision to support continued delivery of the intervention.

**Table 3:** Adaptive delivery processes, original delivery expectations, delivery forms modified, core functions protected, and CFIR mapping

Original delivery plan/SOP expectation	Adaptive delivery process	Description of the adjustment	Delivery form modified	Core function protected	Primary CFIR domain/construct
CHWs were expected to deliver one structured household session per month to the target adolescent, with caregiver involvement, following the monthly module schedule.	Flexible scheduling and revisits	Adjusting home visit timing, conducting revisits, and coordinating with participants to address availability constraints due to schooling, livelihood activities, and mobility.	Visit timing, revisits, participant follow-up arrangements.	Completion of monthly module exposure and target adolescent engagement along with caregiver.	**Outer setting**: recipient needs and resources; implementation process: executing.
CHWs were expected to deliver key module messages using the visual-aided flyer and SOP-guided session flow.	Contextualized reinforcement of complex content	Reinforcing key messages through repetition and locally relevant examples to improve comprehension of complex intervention concepts.	Communication approach, examples used, repetition of explanations.	Key nutrition messages, visual-aided counselling, and participant comprehension.	**Intervention characteristics**: adaptability; **characteristics of individuals**: knowledge and beliefs.
CHWs were expected to deliver standard nutrition messages while encouraging feasible household-level application.	Resource-sensitive counselling adjustment	Aligning dietary recommendations with locally available and affordable foods while preserving core intervention intent.	Locally feasible food examples and substitutions.	Core dietary messages on diversity, healthier choices, and anaemia prevention.	**Outer setting**: recipient needs and resources; intervention characteristics: adaptability.
CHWs were expected to complete assigned household visits within each monthly delivery period.	Workload-responsive micro-planning	Clustering household visits and adjusting schedules to manage competing CHW responsibilities and sustain delivery continuity.	CHW visit scheduling, clustering of nearby households, delivery sequencing.	Intervention dose, continuity of delivery, and SOP-guided session structure.	**Inner setting**: available resources; implementation process: planning and executing.
CHWs were expected to engage the target adolescent and caregiver during household visits and complete monthly module exposure.	Network-supported participant follow-up	Engaging caregivers, neighbours, and local leaders to support participant follow-up and continuity of intervention exposure.	Follow-up pathways, caregiver/community contact mechanisms.	Participant retention, caregiver involvement, and continuity of exposure.	**Outer setting**: external networks; implementation process: engaging.

### Outer setting: Flexible scheduling and revisit mechanisms

Participant availability constrained delivery across implementation periods, requiring CHWs to use flexible scheduling and revisits to support completion of household sessions. Availability was shaped by livelihood activities, school attendance, and temporary mobility, which constrained the ability of CHWs to conduct household sessions as planned.

During the first implementation month (May 2025), rainfall and agricultural labour demands disrupted planned visits and reduced daytime household availability, limiting opportunities for delivery.

Sometimes it rains when we are already on the way to deliver the education message to the households, so we fail to implement that day. (CHW, male, 32 years, first implementation month)

Parents also described how livelihood activities limited their availability during the day, requiring CHWs to adjust visit timing.

Sometimes during the day, we are not at home because we go to the farm or to work, so when they [CHWs] come and miss us, they have to return later. (Parent, female, 41 years, third implementation month)

Relatedly, adolescents also noted that their availability, particularly due to schooling, influenced scheduling of visits.

Sometimes I am at school or away, so they tell my parent they will come back when I am around. (Adolescent, female, 14 years, fourth implementation month)

In response to these challenges, CHWs adjusted by returning later in the day or rescheduling visits to subsequent days to ensure sessions were completed. These scheduling challenges were discussed during supervision, helping CHWs to harmonize revisit practices while maintaining the intended monthly module delivery. CHWs also addressed temporary relocation by coordinating with caregivers and community members to support follow-up, thereby supporting continuity while adjusting visit timing and revisits.

If we reach and find they have gone to the garden, we return in the evening or come back the next day so that we find them at home. (CHW, female, 49 years, fourth implementation month)

### Intervention characteristics and characteristics of individuals: Contextualized reinforcement of complex content

Some intervention topics required repeated explanation and contextualization across implementation periods, particularly where concepts were abstract or not directly observable in daily life. CHWs described difficulties explaining certain topics, especially where adolescents could not easily relate them to everyday experiences.

Both CHWs and the intervention recipients (adolescents and parents) described initial difficulties in understanding some nutrition concepts before repeated explanation.

At first I did not understand some of the lessons even though they were in Lusoga language, but when they kept explaining and using examples we see at home, I started to understand. (Young adult, female, 20 years, third implementation month)

Training on anaemia was hard at first because the adolescents did not know that poor diet quality and worms can make them have little blood in their bodies. (CHW, male, 56 years, seventh implementation month)

When they explain slowly and repeat, our adolescents and myself began to understand better compared to the previous visits. (Parent, female, 47 years, third implementation month)

To address these comprehension challenges, CHWs strengthened communication through repetition, contextualization, and participatory engagement. Feedback on participant understanding was discussed during supervision and used to support more consistent communication approaches across CHWs. CHWs used familiar foods and everyday examples to reinforce understanding across visits, thereby modifying communication approaches while maintaining core nutrition messages and visual-aided counselling.

We started explaining using foods they see every day ‒ like vegetables, beans, and mangoes ‒ and repeated this in later visits until they understood. (CHW, male, 56 years, fourth implementation month)

Supervisory discussions also encouraged CHWs to link messages to adolescents’ lived experiences and increase participation during sessions, supporting continued refinement of communication practices without changing core intervention function.

With time we learned how to involve them more… which made the lessons easier to follow. (CHW, female, 50 years, seventh implementation month)

### Outer setting: Resource-sensitive counselling adjustments

Household economic conditions shaped the feasibility of applying recommended dietary practices across implementation periods, requiring CHWs to tailor counselling examples to locally available and affordable foods. Adolescents described challenges in accessing recommended foods due to financial constraints, highlighting limitations in implementing dietary advice.

They told us to eat eggs and fruits to stay healthy, but sometimes there is no money at home to buy them, so you just eat what is available. (Adolescent, male, 15 years, second implementation month)

Caregivers similarly emphasized reliance on available foods, reflecting household-level constraints that shaped dietary practices.

You may want to have fish or fruits for the children, but when you cannot afford them you just prepare what is there. (Parent, female, 58 years, third implementation month)

Adolescents further reported that household food choices were shaped by financial constraints, where even supportive caregivers could not always prioritize healthier options due to limited resources and competing household needs.

My mum supports us to eat well, but sometimes my father says there is no money for those foods. (Adolescent, female, 17 years, seventh implementation month)

Through supervisory discussions, participant feedback on limited access to recommended foods was reviewed, and CHWs were guided to align dietary advice with locally available resources while maintaining core nutrition messages. This supported a more consistent approach to resource-sensitive counselling across CHWs without changing the intended dietary messages. CHWs emphasized affordable and available food options by modifying food examples and substitutions while preserving the intent of dietary recommendations.

We explain that even if they cannot buy expensive foods, they can use greens from the garden, beans, and groundnuts so the child still eats appropriately. (CHW, female, 32 years, seventh implementation month)

### Inner setting and implementation process: Workload-responsive micro-planning

Competing CHW responsibilities and household scheduling constraints affected delivery across implementation periods, requiring micro-planning to sustain household visits within the intended monthly delivery cycle. CHWs reported competing responsibilities that affected their ability to maintain planned visit schedules.

CHWs described how overlapping responsibilities limited their ability to conduct household visits as planned.

Sometimes we have many activities in the community at the same time… you are expected to attend a meeting, support another health program, and still visit households. (CHW, male, 61 years, first implementation month)

CHWs also highlighted situations where they were required to be present in multiple locations at the same time, which constrained delivery.

You can be called for immunization mobilisation or meetings… yet you also have homes to visit, so sometimes you find you are expected in two places at once. (CHW, male, 45 years, third implementation month)

Caregivers also described how visit timing needed to be adjusted to fit household routines, noting that subsequent visits were better aligned with their availability after prior discussion with CHWs.

During first visit, the CHW came when we were busy, but later she arranged and came at a better time when we were available. And for the subsequent visits, she started consulting me to know when both my adolescents and myself will be available before she could come. (Parent, female, 44 years, third implementation month)

As an adaptive response, CHWs were guided to reorganize delivery through micro-scheduling strategies to sustain coverage under constrained conditions. Supervisory discussions helped CHWs share and harmonize practical scheduling approaches, including clustering nearby households and adjusting visit timing. These adjustments modified visit scheduling and sequencing while seeking to maintain intervention dose and continuity of delivery.

We started grouping two homes that are near each other and visiting them on the same day… that way we save time and make sure we don’t miss the lessons. (CHW, female, 50 years, fifth implementation month)

### Outer and inner setting: network-supported participant follow-up

Participant absence and temporary mobility affected continuity of delivery across implementation periods, requiring CHWs to draw on household and community networks to support follow-up. CHWs described engaging neighbours and local leaders to locate participants and support follow-up when adolescents were temporarily unavailable.

Sometimes you reach the home and find the adolescent has gone to stay with an aunt in another village. I would ask neighbours or the village local council to help locate them and follow up so they do not miss the lessons. (CHW, male, 56 years, fourth implementation month)

Through supervisory discussions, these follow-up approaches were refined to strengthen coordination with caregivers and local council one leaders across implementation periods. Caregivers also reported active engagement in supporting continuity of intervention messages within households between visits.

Even when the CHW is not around, we continue reminding the children about what they were taught and try to follow those practices at home. (Parent, male, 53 years, sixth implementation month)

### Integrated adaptation–fidelity interplay during implementation

To examine adaptation–fidelity interplay during implementation, fidelity findings were interpreted alongside qualitative accounts of contextual influences, delivery challenges, supervisory reflection, and adaptive delivery adjustments (Table 4). Fidelity scores were generally high across implementation periods, with only modest variation across domains. This analysis identified how delivery challenges were discussed during supervision and how adaptive delivery adjustments were considered in response to contextual influences across implementation periods.

**Table 4:** Integrated summary of contextual influences, supervisory reflection, adaptive delivery adjustments, and fidelity findings across implementation periods

Contextual influences and delivery challenges	Supervisory reflection and guidance	Adaptive delivery adjustments	Fidelity findings and interpretation
Participant availability constraints, including rainfall, agricultural work, school attendance, and temporary mobility, created risks of missed or delayed household sessions.	Review of missed or delayed sessions; guidance on revisits, flexible scheduling, and caregiver follow-up.	Use of revisits, evening or alternative-day scheduling, and coordination with caregivers and community members to support participant follow-up.	Dose delivered remained high across implementation periods, with only modest variation. Qualitative accounts indicate that flexible scheduling and revisits were used to manage availability constraints while preserving the monthly module delivery intent.
Some nutrition concepts, particularly those perceived as abstract or not directly observable, required repeated explanation and contextualization.	Review of comprehension challenges; guidance on repetition, locally relevant examples, and participatory communication.	Use of familiar foods, repeated explanations, and participatory facilitation to reinforce understanding across visits.	Quality of delivery remained high across implementation periods, and participant responsiveness showed only modest variation. Qualitative accounts indicate that localized communication adjustments were used to support comprehension without changing the intended intervention content.
Household economic constraints limited the feasibility of applying some dietary recommendations, particularly where recommended foods were perceived as costly or unavailable at household level.	Review of feasibility concerns; guidance on aligning dietary counselling with locally available and affordable foods.	Modification of food examples and substitutions to reflect locally available, affordable options while preserving core dietary messages.	Program differentiation scores were high when assessed during mid and late implementation. Qualitative accounts indicate that counselling adjustments focused on delivery examples and feasibility, rather than changing the core dietary messages.
Competing CHW responsibilities and household scheduling constraints affected the ability to maintain planned visit schedules.	Review of workload and scheduling constraints; guidance on clustering visits, sequencing nearby households, and adjusting visit timing.	Clustering of nearby household visits and adjustment of delivery sequencing to improve efficiency under constrained conditions.	Adherence remained high and stable across implementation periods. Qualitative accounts suggest that micro-planning was used to manage workload and scheduling constraints while sustaining planned household delivery.
Participant absence and temporary mobility interrupted continuity of engagement for some households.	Review of follow-up challenges; guidance on engaging caregivers, neighbours, and local leaders where needed.	Coordination with caregivers, neighbours, and local leaders to support participant follow-up and continuity of exposure.	Dose received and participant responsiveness remained high, with modest variation across implementation periods. Qualitative accounts indicate that network-supported follow-up was used to manage participant absence and support continuity of exposure.

Note. Note. The table presents integrated analysis of quantitative fidelity findings and qualitative accounts; it does not estimate the effects of specific adaptive delivery adjustments on fidelity outcomes

## Discussion

This study assessed implementation fidelity over time and examined how contextual influences and supervision-guided adaptive delivery adjustments shaped delivery processes during the nine-month implementation of a CHW-led intervention in rural Uganda. By integrating longitudinal fidelity assessment with repeated qualitative evidence on implementation processes, the findings indicate that fidelity and adaptive delivery adjustments were not necessarily competing priorities, but were managed through structured supervisory reflection. Supportive supervision provided a forum for discussing delivery challenges, reviewing practical solutions, and agreeing on context-appropriate adaptive delivery adjustments while preserving core intervention functions.

Fidelity scores remained generally high throughout the implementation period, with only modest variation across domains. Delivery challenges were nevertheless evident, including participant availability constraints, comprehension difficulties, household resource limitations, competing CHW responsibilities, and scheduling demands. Similar variability in dose delivered and participant responsiveness has been documented in community-based interventions [13, 22]. In the present study, repeated delivery experience and supportive supervision facilitated adjustments in scheduling, communication, and counselling approaches, suggesting that fidelity in routine CHW-led delivery should be understood as a process shaped by changing implementation conditions rather than as a static outcome assessed at a single time point [36]. For practice, this underscores the importance of building flexibility into delivery protocols, including planned revisits, adaptive scheduling, and mechanisms to sustain targeted implementation under varying contextual demands.

Supportive supervision evolved as a central mechanism through which adaptive delivery adjustment and fidelity were aligned over time. Rather than functioning solely as a monitoring tool, supervision provided a structured platform for reflection, feedback, and guided adjustment. Through this process, observed delivery challenges were reviewed and used to inform subsequent adjustments in delivery practices. This is consistent with evidence from community health systems where supervision supports learning and quality improvement [14] and facilitates alignment of delivery practices in CHW programs [13]. For policy and health systems strengthening, these findings point to the need to invest in supportive supervision models that integrate real-time feedback, problem-solving, and decision support, rather than relying on compliance-oriented supervision alone.

The integrated findings provide a process-oriented account of how fidelity and adaptive delivery adjustments were managed during routine implementation. It shows how implementation experiences informed supervisory reflection, and supervisory reflection supported context-based adaptive delivery adjustments intended to preserve core intervention functions. Delivery forms were adjusted to fit contextual realities while core intervention functions remained consistent across implementation. The longitudinal nature of this process indicates that alignment between delivery adjustments and fidelity evolves over time, rather than being achieved at a single point [36]. For research, this underscores the value of longitudinal mixed-methods approaches that capture how implementation processes unfold and highlights the need for analytic frameworks that examine relationships between context, supervision, adaptive delivery adjustments, and fidelity in real-world settings.

Lastly, the findings are analytically transferable rather than statistically generalizable. The adaptive delivery adjustments observed in this study were shaped by household routines, school attendance, seasonal livelihood activities, CHW workload, and local food-resource constraints in rural eastern Uganda. However, the broader implementation lessons may be relevant to other CHW-led, household-level interventions in low-resource settings where frontline workers must balance implementation fidelity to core intervention functions with flexibility in delivery. In such settings, clear specification of core intervention functions, modifiable delivery forms, and supervision mechanisms can help guide context-responsive implementation without treating all delivery adjustments as fidelity threats.

### Study strengths and limitations

The longitudinal mixed-methods design enabled assessment of fidelity across implementation periods, and examination of how adaptive delivery was managed under routine conditions. The integration of quantitative fidelity measures with repeated qualitative data from CHWs, adolescents, and caregivers strengthened the credibility of findings and provided insight into how delivery challenges, supervision, and adaptive delivery adjustments were connected across implementation periods. Fidelity data were collected prospectively across early, mid, and late implementation, reducing recall bias and enabling temporal comparison of fidelity. Embedding the study within routine CHW delivery systems further enhances the relevance of the findings to real-world implementation contexts.

However, fidelity assessments were periodic rather than continuous and may not have captured all session-level variation; this was mitigated through repeated observations and triangulation with qualitative data from implementers, intervention recipients, and parents or guardians. Fidelity observations were conducted by supervisors involved in implementation, which may have introduced observer or social desirability bias; standardized checklists and unannounced visits were used to minimize this risk. Although qualitative data were collected repeatedly, FGDs with AYAs and parents involved different participants across rounds; therefore, findings reflect evolving implementation experiences across participant groups rather than individual-level change. Purposive sampling and triangulation across participant groups helped address this limitation. Because adaptive delivery adjustments were identified through qualitative accounts and supervision-linked interpretation, the study cannot estimate the independent effects of specific adjustments on fidelity outcomes. Instead, it provides an integrated longitudinal account of supervision, adaptive delivery, and fidelity under routine implementation conditions.

## Conclusion

This study provides longitudinal empirical evidence that adaptive delivery and implementation fidelity can be managed as interdependent implementation processes rather than competing priorities. In this CHW-led adolescent nutrition intervention, fidelity monitoring, supportive supervision, and guided problem-solving created a feedback process through which adaptive delivery adjustments were made while preserving core intervention functions. These findings highlight the value of structured supervision systems in guiding context-responsive delivery adjustments and maintaining fidelity in routine community health delivery.

## Supplementary Information

Below is the link to the electronic supplementary material.

Supplementary Material 1

Supplementary Material 2

Supplementary Material 3

Supplementary Material 4

Supplementary Material 5

## Data Availability

The de-identified quantitative fidelity dataset supporting the findings of this study is provided as Supplementary Material 5. Excerpts from qualitative transcripts sufficient to support the analytic claims are presented in the manuscript. Full de-identified qualitative transcripts are not publicly available due to ethical and confidentiality considerations but may be made available from the corresponding author on reasonable request, subject to applicable ethics and data protection requirements.
