# Supplementary material for: Balancing adaptive delivery and implementation fidelity over time: a longitudinal mixed-methods study of a community health worker–led adolescent nutrition intervention in rural Uganda
Source: Implement Sci Commun. 2026 Aug 1;7:218. doi: 10.1186/s43058-026-01072-0 (PMC13721700; doi:10.1186/s43058-026-01072-0)
Supplement: Supplementary file 1 [file 43058_2026_1072_MOESM2_ESM.docx]

**FOCUS GROUP DISCUSSION GUIDE ‒ COMMUNITY HEALTH WORKERS (Implementers)**

**Study:** Implementation of the adolescent nutrition intervention
**Objective:** To explore evolving contextual, organizational, and individual determinants (barriers and facilitators) influencing implementation, and adaptations over time, guided by the CFIR.
**Participants:** CHWs delivering the adolescent nutrition intervention in Mayuge, Eastern Uganda
**Duration:** 60–75 minutes

**General Information**

Sub-county: ___________________ Parish: ___________________ Village: ___________________
Date: __________ Moderator: __________ Note-taker: __________
Session No.: _____ No. of CHWs: _____
Start time: __________ End time: __________

**Introduction**

- Purpose of discussion; confidentiality assured
- Participation voluntary; permission to audio-record
- Respect and equal participation encouraged

**Discussion Questions**

**1. Intervention characteristics**

1. How would you describe your role in the intervention program?
2. What do you think about the design of the intervention and how it works in homes?
3. How useful and practical are the materials provided (visuals, flyers, guides)?
4. Which parts of the messages are easy or difficult to explain?

**2. Outer setting – Community and household influences**

5. How are adolescents and parents responding to the home visits?
6. What community or household issues affect participation or cooperation?

**3. Inner setting – Work environment, resources, support**

7. How are you integrating intervention activities into your routine CHW responsibilities?
8. What supports your ability to deliver the activities well?
9. What structural challenges have you faced?

**4. Characteristics of individuals – Capacity, confidence, motivation**

10. How confident do you feel in implementing each activity?
11. What personal strategies have helped you overcome difficulties?
12. What motivates you to continue delivering intervention?

**5. Implementation process – Execution, reflection, adaptation**

13. What has worked well in delivering the sessions?
14. What has not worked well? Why?
15. Have there been any changes in how you deliver the sessions over time?
    What made those changes helpful or unhelpful?
16. What support do you need to improve your work?
17. Any additional suggestions or concerns?

**Closing**

Thank CHWs and reassure confidentiality.

**Moderator Summary**

Successes / What worked well: ___________________________________
Facilitators: _________________________________________________
Challenges / What did not work: _________________________________
Reasons for challenges: _________________________________________
Support needs identified: _______________________________________
Actions and follow-up: _________________________________________

**FOCUS GROUP DISCUSSION GUIDE – ADOLESCENTS AND YOUNG ADULTS**

**CFIR-Informed Focus Group Discussion Guide – Adolescents/Young Adults**
**Study:** Implementation of the Adolescent Nutrition Intervention
**Objective:** To explore adolescents’ experiences, acceptability, contextual influences, and evolving determinants of participation in the intervention, guided by CFIR.
**Participants:** Adolescents/Young adults aged 10–24 years
**Duration:** 60–75 minutes

**General Information**

Sub-county: ___________________ Parish: ___________________ Village: ___________________
Date: __________ Moderator: __________ Note-taker: __________
FGD No.: _____ No. of Participants: _____
Start time: __________ End time: __________

**Introduction**

- Purpose of discussion; confidentiality and voluntary participation
- Respect for all opinions; permission to audio-record

**Discussion Questions**

**1. Community needs and experience**

1. Before this intervention, what did you know about healthy eating?
2. What nutrition problems do adolescents here commonly face?
3. How did you feel when you were invited to participate?

**2. Views on the CHW sessions and messages**

4. What do you think about the home-visit education sessions?
5. How do the pictures and flyers help you understand the messages?
6. Which topics did you find most/least useful? Why?
7. Were any parts unclear or not relevant?

**3. Behavior change and influences**

8. Have you tried any practices you learned? Please give examples.
9. What helps you follow the advice from CHWs?
10. What makes it difficult to follow the messages?

**4. Household support**

11. How do your family members respond to the visits?
12. Do they support or discourage you in making changes? How?

**5. Delivery experience and adaptation**

13. How do you feel about the way CHWs interact with you?
14. Do sessions happen at a suitable time?
15. Have you noticed any changes in how CHWs conduct sessions over time?
    Did those changes make participation easier or harder?

**6. Suggested improvements**

16. If you were in charge, what would you change or improve?
17. Would you encourage friends to participate? Why or why not?
18. Any other comments or questions?

**Closing**

Thank participants and remind confidentiality.

**Moderator Summary**

What worked well: _____________________________________________
Barriers/Challenges: ___________________________________________
Facilitators: _________________________________________________
Suggested improvements: _______________________________________
Action points: ________________________________________________

**FOCUS GROUP DISCUSSION GUIDE – PARENTS / HOUSEHOLD MEMBERS**

**CFIR-Informed Focus Group Discussion Guide – Parents/Household Members**
**Study:** Implementation of the Adolescent Nutrition Intervention
**Objective:** To explore household-level determinants (barriers, facilitators), and evolving implementation experiences, guided by CFIR.
**Participants:** Parents/caregivers of participating adolescents and young adults (AYAs)
**Duration:** 60–75 minutes

**General Information**

Sub-county: ___________________ Parish: ___________________ Village: ___________________
Date: __________ Moderator: __________ Note-taker: __________
FGD No.: _____ No. of Participants: _____
Start time: __________ End time: __________

**Introduction**

Purpose of discussion; confidentiality; voluntary participation; permission to audio-record.

**Discussion Questions**

**1. Community needs and relevance**

1. What nutrition challenges do AYAs here commonly face?
2. What made you accept CHWs visiting your home?

**2. Usefulness and clarity of the intervention**

3. How useful are the messages shared during visits?
4. What do you think about the visual pictures and flyers left with you?

**3. Household responses and practices**

5. Have you made any changes at home because of the sessions? Please give examples.
6. Are there any cultural beliefs or customs that make it difficult to follow the messages?

**4. Household priorities and support**

7. What challenges do you face in supporting healthier eating?
8. Who usually decides what is bought and cooked at home?

**5. Experience of CHW engagement and adaptation**

9. How do you feel about the way CHWs interact with your family?
10. How do other community members view this intervention?
11. Have you noticed any changes in how CHWs deliver the visits over time?
    Did those changes make participation easier or harder?

**6. Improvement suggestions**

12. What helps you maintain the nutrition practices being encouraged?
13. What changes would make this program more useful for families?
14. Any final suggestions or comments?

**Closing**

Thank participants; reassure confidentiality.

**Moderator Summary**

What worked well: _____________________________________________
Facilitators: _________________________________________________
Challenges: _________________________________________________
Reasons for challenges: ________________________________________
Suggestions: ________________________________________________
Action points & follow-up: _____________________________________
