# Supplementary material for: Balancing adaptive delivery and implementation fidelity over time: a longitudinal mixed-methods study of a community health worker–led adolescent nutrition intervention in rural Uganda
Source: Implement Sci Commun. 2026 Aug 1;7:218. doi: 10.1186/s43058-026-01072-0 (PMC13721700; doi:10.1186/s43058-026-01072-0)
Supplement: Supplementary file 2 [file 43058_2026_1072_MOESM3_ESM.docx]

**Supplementary Material 3.** Summary coding tree

**Manuscript title:** Balancing adaptive delivery and implementation fidelity over time: a longitudinal mixed-methods study of a community health worker-led adolescent nutrition intervention in Uganda

**Purpose:** This summary coding tree shows how main code categories and illustrative subcodes were organized into themes describing adaptive delivery processes during implementation. Subcodes are illustrative and were used to support interpretation of qualitative findings across participant groups and implementation periods.

| **Main code category** | **Illustrative subcodes** | **Resulting theme** |
| --- | --- | --- |
| Participant and household availability | School attendance; farm work and livelihood activities; rainfall; temporary absence or mobility; missed household contacts; caregiver availability | Flexible scheduling and revisits |
| Comprehension and communication challenges | Difficulty understanding abstract nutrition concepts; repeated explanations; locally familiar examples; participatory discussion; use of visual-aided materials | Contextualized reinforcement of complex content |
| Household resource constraints | Food affordability; local food availability; household food decision-making; feasible substitutions; adapting examples to household food resources | Resource-sensitive counselling adjustment |
| CHW workload and delivery planning | Competing CHW responsibilities; scheduling constraints; clustering nearby households; sequencing visits; rescheduling to maintain monthly coverage | Workload-responsive micro-planning |
| Follow-up and continuity support | Caregiver support; neighbours; local leaders; participant mobility; between-visit reminders; household support for continued exposure | Network-supported participant follow-up |
