# Supplementary material for: Balancing adaptive delivery and implementation fidelity over time: a longitudinal mixed-methods study of a community health worker–led adolescent nutrition intervention in rural Uganda
Source: Implement Sci Commun. 2026 Aug 1;7:218. doi: 10.1186/s43058-026-01072-0 (PMC13721700; doi:10.1186/s43058-026-01072-0)
Supplement: Supplementary file 3 [file 43058_2026_1072_MOESM4_ESM.docx]

**Supplementary Material 4. Completed COREQ checklist**

**Manuscript title:** Balancing adaptive delivery and implementation fidelity over time: a longitudinal mixed-methods study of a community health worker-led adolescent nutrition intervention in rural Uganda

| **No.** | **Item** | **Guide questions/description** | **Reporting status/details** | **Reported in manuscript section** |
| --- | --- | --- | --- | --- |
| **Domain 1: Research team and reflexivity** | | | | |
| **Personal characteristics** | | | | |
| 1 | Interviewer/facilitator | Which author/s conducted the interview or focus group? | Reported. FGDs were conducted by a three-member research team: two female researchers and one male researcher (TB), who was also the field implementation supervisor. | Data collection procedures - Adaptive delivery processes |
| 2 | Credentials | What were the researcher's credentials? (e.g., PhD, MD) | Reported. All three FGD team members held Master of Public Health degrees. | Data collection procedures - Adaptive delivery processes |
| 3 | Occupation | What was their occupation at the time of the study? | Reported. At the time of the study, the FGD team members were Research Associates at Makerere University School of Public Health. | Data collection procedures - Adaptive delivery processes |
| 4 | Gender | Was the researcher male or female? | Reported. The facilitation team included two female researchers and one male researcher. | Data collection procedures - Adaptive delivery processes |
| 5 | Experience and training | What experience or training did the researcher have? | Reported. The team was fluent in English and Lusoga and had prior experience in implementation research and qualitative data collection; regular debriefing meetings with senior researchers supported consistency and data quality. | Data collection procedures - Adaptive delivery processes |
| **Relationship with participants** | | | | |
| 6 | Relationship established | Was a relationship established prior to study commencement? | Reported. Researchers were not members of the study communities and had no prior personal relationships with participants. TB interacted with CHWs during implementation supervision. | Data collection procedures - Adaptive delivery processes |
| 7 | Participant knowledge of interviewer | What did participants know about the researcher? | Reported. Researchers introduced themselves as public health professionals conducting a study on intervention delivery experiences; objectives, confidentiality, and voluntary participation were explained before consent or assent. | Data collection procedures - Adaptive delivery processes; Ethical approval and consent to participate |
| 8 | Interviewer characteristics | What characteristics were reported about the interviewer/facilitator? | Reported. The manuscript states that researchers were fluent in English and Lusoga, familiar with rural Eastern Ugandan contexts and adolescent nutrition issues, and not members of the study communities; TB was also the field implementation supervisor. | Data collection procedures - Adaptive delivery processes |
| **Domain 2: Study design** | | | | |
| **Theoretical framework** | | | | |
| 9 | Methodological orientation and theory | What methodological orientation was stated to underpin the study? | Reported. The study is described as a longitudinal mixed-methods implementation study using a hybrid inductive-deductive thematic approach, informed by CFIR and Carroll's fidelity framework. | Study design; Conceptual framework; Data management and analysis - Adaptive delivery processes |
| **Participant selection** | | | | |
| 10 | Sampling | How were participants selected? | Reported. Purposive sampling was guided by participant role as implementer or recipient, exposure to the intervention, and ability to provide detailed accounts of delivery processes. | Study population and sampling |
| 11 | Method of approach | How were participants approached? | Reported. CHWs were invited as the intervention implementers. AYAs and parents/guardians were approached through household visits; with community contact facilitated (but not influenced) by CHWs and local council one chairpersons. | Study population and sampling; Data collection procedures - Adaptive delivery processes |
| 12 | Sample size | How many participants were in the study? | Reported. A total of 60 unique qualitative participants contributed: 30 AYAs, 20 parents/guardians, and 10 CHWs. | Study population and sampling; Results - Socio-demographic characteristics; Table 1 |
| 13 | Non-participation | How many people refused to participate or dropped out? Reasons? | Reported transparently. All ten CHWs consented to participate. All AYAs and parents contacted also consent and or assented to participate. None dropped out. | Study population and sampling |
| **Setting** | | | | |
| 14 | Setting of data collection | Where was the data collected? | Reported. FGDs were conducted in private, neutral venues, such as schools or health centres, at times convenient to participants. | Data collection procedures - Adaptive delivery processes |
| 15 | Presence of non-participants | Was anyone else present besides the participants and researchers? | Reported. Only FGD participants and research team members were present during discussions; CHWs and local leaders supported recruitment and scheduling but did not attend the FGDs. | Data collection procedures - Adaptive delivery processes |
| 16 | Description of sample | What are the important characteristics of the sample? | Reported. Participant characteristics are summarized by participant group, age, schooling status, education, household size, marital status, disability, and employment. | Results - Socio-demographic characteristics; Table 1 |
| **Data collection** | | | | |
| 17 | Interview guide | Were questions, prompts, guides provided by the authors? Was it pilot tested? | Reported. Semi-structured FGD guides informed by CFIR are provided as Supplementary Material 2. Guides were pretested among non-participants in Iganga district to assess clarity, cultural appropriateness, and question flow; feedback informed minor revisions to wording and sequencing. | Data collection procedures - Adaptive delivery processes; Supplementary Material 2 |
| 18 | Repeat interviews | Were repeat interviews carried out? | Reported. CHW FGDs followed a panel design involving the same ten CHWs across repeated rounds. AYA and parent/guardian FGDs involved different participants across selected rounds. | Study population and sampling; Data collection procedures - Adaptive delivery processes |
| 19 | Audio/visual recording | Did the research use audio or visual recording to collect the data? | Reported. All FGDs were audio-recorded using digital voice recorders with informed consent. | Data collection procedures - Adaptive delivery processes |
| 20 | Field notes | Were field notes made during and/or after the interview or focus group? | Reported. Field notes were taken during or immediately after FGDs to capture non-verbal cues, contextual observations, and emerging issues. | Data collection procedures - Adaptive delivery processes; Data management and analysis - Adaptive delivery processes |
| 21 | Duration | What was the duration of the interviews or focus groups? | Reported. Each FGD lasted 60-90 minutes. | Data collection procedures - Adaptive delivery processes |
| 22 | Data saturation | Was data saturation discussed? | Reported. Sampling and data collection proceeded iteratively until sufficient depth and variation in perspectives were achieved across participant groups. | Study population and sampling |
| 23 | Transcripts returned | Were transcripts returned to participants for comment and/or correction? | Reported. Transcripts were not returned to participants for comment or correction. Accuracy was supported through audio recording, verbatim transcription, translation cross-checks, and review of selected transcripts against original audio recordings. | Data management and analysis - Adaptive delivery processes |
| **Domain 3: Analysis and findings** | | | | |
| **Data analysis** | | | | |
| 24 | Number of data coders | How many data coders coded the data? | Reported. Each transcript was coded independently by at least two research team members, with discrepancies resolved through consensus discussions involving three analysts. | Data management and analysis - Adaptive delivery processes |
| 25 | Description of the coding tree | Did authors provide a description of the coding tree? | Reported. A summary coding tree is provided as Supplementary Material 3. | Data management and analysis - Adaptive delivery processes; Supplementary Material 3 |
| 26 | Derivation of themes | Were themes identified in advance or derived from the data? | Reported. Open coding was first used to identify adaptive delivery processes, delivery forms modified, and whether core functions were preserved; CFIR domains and selected constructs were then used to organize and interpret these processes. | Data management and analysis - Adaptive delivery processes |
| 27 | Software | What software was used to manage the data? | Reported. NVivo version 12 was used for qualitative analysis. | Data management and analysis - Adaptive delivery processes |
| 28 | Participant checking | Did participants provide feedback on the findings? | Reported. Participant checking of final themes was not conducted. Credibility was strengthened through field notes, regular debriefing, team-based coding, consensus discussions, triangulation across CHWs, AYAs, and parents/guardians, and use of illustrative quotations from different participant groups and implementation periods. | Data management and analysis - Adaptive delivery processes; Study strengths and limitations |
| **Reporting** | | | | |
| 29 | Quotations presented | Were participant quotations presented to illustrate the themes/findings? Was each quotation identified? | Reported. Quotations from CHWs, AYAs, and parents/guardians are presented in the Results section and identified by participant category, sex, age, and implementation period. | Results - Adaptive delivery processes and preservation of core intervention functions |
| 30 | Data and findings consistent | Was there consistency between the data presented and the findings? | Reported. Themes are supported by illustrative quotations and summarized in relation to contextual influences, delivery forms modified, and core intervention functions protected. | Results - Adaptive delivery processes and preservation of core intervention functions; Table 3 |
| 31 | Clarity of major themes | Were major themes clearly presented? | Reported. Five major themes are presented: flexible scheduling and revisits; contextualized reinforcement of complex content; resource-sensitive counselling adjustment; workload-responsive micro-planning; and network-supported participant follow-up. | Results - Adaptive delivery processes and preservation of core intervention functions; Table 3 |
| 32 | Clarity of minor themes | Is there a description of diverse cases or discussion of minor themes? | Reported. Subcodes within each theme capture variation by participant group, implementation period, delivery challenge, and delivery form modified; these are summarized in the coding tree (Supplementary Material 3) and illustrated through quotations in the Results. | Results - Adaptive delivery processes and preservation of core intervention functions; Supplementary Material 3 |

**Note.** This checklist reports where each COREQ item is addressed in the manuscript and supplementary materials. Transparent reporting of procedures not undertaken, such as transcript return and participant checking, is included where applicable.
